# Identifying multiscale spatio-temporal patterns in human mobility using manifold learning

**DOI:** 10.7717/peerj-cs.276

**Published:** 2020-06-15

**Authors:** James R. Watson, Zach Gelbaum, Mathew Titus, Grant Zoch, David Wrathall

**Affiliations:** College of Earth, Ocean and Atmospheric Sciences, Oregon State University, Corvallis, OR, USA

**Keywords:** Complex systems, Manifold learning, Human mobility, Dimension reduction, Prediction, Geographic information science, Multiscale, Emergence, Wavelet, Networks

## Abstract

When, where and how people move is a fundamental part of how human societies organize around every-day needs as well as how people adapt to risks, such as economic scarcity or instability, and natural disasters. Our ability to characterize and predict the diversity of human mobility patterns has been greatly expanded by the availability of Call Detail Records (CDR) from mobile phone cellular networks. The size and richness of these datasets is at the same time a blessing and a curse: while there is great opportunity to extract useful information from these datasets, it remains a challenge to do so in a meaningful way. In particular, human mobility is multiscale, meaning a diversity of patterns of mobility occur simultaneously, which vary according to timing, magnitude and spatial extent. To identify and characterize the main spatio-temporal scales and patterns of human mobility we examined CDR data from the Orange mobile network in Senegal using a new form of spectral graph wavelets, an approach from manifold learning. This unsupervised analysis reduces the dimensionality of the data to reveal seasonal changes in human mobility, as well as mobility patterns associated with large-scale but short-term religious events. The novel insight into human mobility patterns afforded by manifold learning methods like spectral graph wavelets have clear applications for urban planning, infrastructure design as well as hazard risk management, especially as climate change alters the biophysical landscape on which people work and live, leading to new patterns of human migration around the world.

## Introduction

Human mobility is a fundamental part of how individuals, households and communities organize to meet every-day needs, and to respond to infrequent risks and shocks like economic instability and environmental hazards. Human mobility is multiscale in nature (Song et al., 2010a), that is for any given type of mobility, such as commuting, seasonal migration or holiday travels, individuals move as part of social collectives of varying size and interconnectivity, which span different magnitudes of spatial and temporal scale. Human mobility also has multiple spatio-temporal modes of variability: people go to work each day, they go on holiday during specific programed periods within the year, they may migrate before and after key agricultural seasons, or they may evacuate during floods or other environmental hazards (Widhalm et al., 2015). For these reasons and others, it is a continuing challenge to identify, categorize and anticipate the various patterns of human mobility (Simini et al., 2012). Anticipating and planning for human mobility is a non-trivial task for organizations whose core functions provide critical services to and address the needs of moving people, such as urban planning and transport agencies, disaster first-responders and international aid organizations (Jiang, Ferreira & Gonzalez, 2017).

To overcome these challenges and generate fundamental insight on human mobility, novel data generated by users of the digital infrastructure (e.g., mobile phone subscribers) is now being used. So-called Big Data, routinely collected from a range of sources, including social platforms like Twitter, Flickr and Facebook (Barbosa et al., 2018) and most notably the explosion of mobile phone usage throughout the world, provides rich information on users’ locations through time (Giannotti et al., 2011). Mobile network operators collect records of their users’ calling patterns, a type of data called Call Detail Records (CDR), which include the location of the receiving tower where each voice call or text message is made, as well as the location of the recipient. Over time, each user’s calling patterns can be used to reconstruct a detailed record of their location history. The collective mobility history of all users’ movements through time provides insight on total population flows between all cellular network locations during any specified period of time. This enables the study of users’ behaviors at very high spatiotemporal resolution over both local and system-level spatial scales at time scales of minutes to months to years (Song et al., 2010b). As each phone is embedded within an existing social fabric, CDR allow the analysis of the changing structure of social organization as people (i.e., individuals, social networks, communities, religious and ethnic groups, etc.) respond to a diversity of stimuli. CDR data has been used for urban planning (Becker et al., 2011), and to describe and evaluate commuting (Iqbal et al., 2014) and environmental displacement resulting from earthquakes and hurricanes (Lu et al., 2016; Lu, Bengtsson & Holme, 2012). They have also been used to evaluate mobility as a vector for the spread of infectious diseases such as ebola (Wesolowski et al., 2014a, 2014b; Balcan et al., 2009; Belik, Geisel & Brockmann, 2011).

CDR data offer a vastly clearer and more detailed description of the communication and mobility behaviors of people as they go about their daily lives, compared to traditional data on human mobility, such as surveys and censuses, which are collected at relatively coarse time-scales, that is, months and years (Bell & Ward, 2000). CDRs are compiled by network operators principally for the purposes of billing customers for their use of the network, not for scientific analysis; therefore, the main challenge with analyzing CDR is the size, complexity and richness of datasets. CDR are inherently high dimensional and noisy (Chen & Zhang, 2014), and quantitative analyses must incorporate dimensional reduction and denoising approaches. Once done, the remaining challenge is retrieving a relevant signal from the data at the appropriate spatial and temporal scale for each specific mobility pattern (Song et al., 2010b).

**Figure 1:**
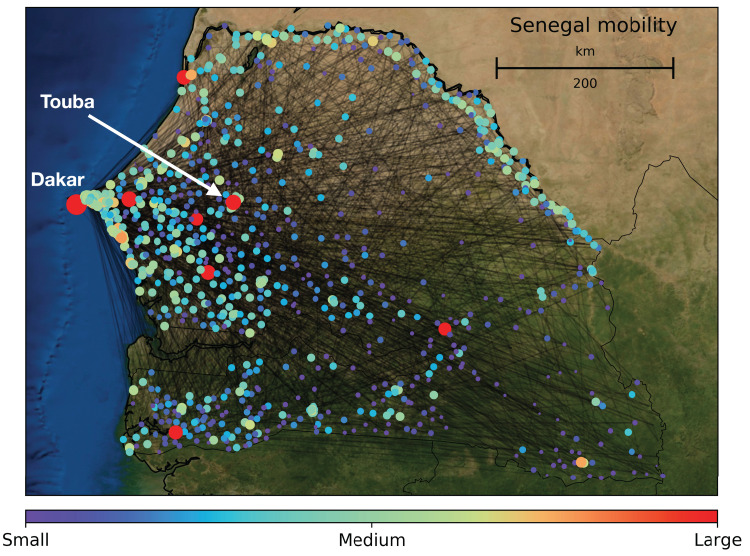
Map of Senegal with major cellular communication towers: these are the nodes in our human mobility networks. Nodes are color coded and sized by population density and edges connecting them highlight the density and complexity of human communication and mobility networks. These networks are changing over time, and in Senegal there are many large-scale mobility events, relating to religious events (often held at Touba, the focal city of this study) as well as to seasonal changes in weather and agriculture.

Here, we describe a data-driven approach to CDR analysis that explicitly addresses the multiscale nature of the mobility patterns embedded in the data and reflected in the system under study. We analyzed CDR from users of the Orange/Sonatel cellular network, collected in Senegal between 1 January and 31 December 2013 (see Fig. 1 for an illustration of mobility in Senegal for a given day). De-identified data entries included information on the time of the call, the mobile phone tower used and the duration of call. To identify and characterize different spatio-temporal modes of human mobility captured in CDR, we developed a novel computational approach based on spectral graph wavelets, an extension of classical wavelet analysis to the setting of networks. There are now numerous examples of where CDR data has been used to understand patterns of human mobility (Jarv, Ahas & Witlox, 2014; Calabrese et al., 2013; Dobra, Williams & Eagle, 2015), including those that also explicitly address multiscale patterns (Phithakkitnukoon, Smoreda & Olivier, 2012) using methods from statistical physics (Lambiotte et al., 2008; Simini et al., 2012). Here, we have made new mathematical advances to spectral graph wavelets to improve upon these state of the art approaches to the multiscale decomposition of CDR data. In doing so we were able to identify and characterize various multiscale spatio-temporal patterns of human mobility in Senegal for the year 2013.

To demonstrate the utility of our approach we focused on dynamic multiscale mobility patterns to/from a specific city in Senegal—Touba, a market town and religious center in Senegal’s agricultural breadbasket. Our goal was to identify and characterize the different spatio-temporal patterns of human mobility that contrast in overall spatial scale and temporal duration. We focus on inter-city forms of mobility to/from Touba, including seasonal migration relating to changes in agriculture and a mobile labor force, as well as punctuated patterns relating to calendar festivals, local elections, or religious holidays. The identification and interpretation of these spatio-temporal scales and patterns of human mobility is of value to the ultimate goal of extracting key dynamics from complex adaptive systems in general.

## Methods

### Human mobility data

To analyze the multiscale nature of human mobility, we developed a new approach to spectral graph wavelets which we then applied to de-identified, pre-processed extracts of CDR from the Orange/Sonatel mobile network in Senegal generated between January 1 and December 31, 2013. These data were obtained from the Orange Telecom Data for Development Challenge (D4D) and are highly sensitive. As a consequence they cannot be made publicly available, but can be obtained by contacting Orange. These data include a number of variables on individual mobility behaviors/locations for 300,000 randomly sampled users on a rolling 2-week basis. These data were arranged into a pairwise origin-destination network whose vertices, or nodes, are cell towers and whose edges are weighted by the volume of phones moving/flowing between each tower pair for each 24-h period over the entire year of 2013. To highlight inter-city mobility, we first aggregated the cell towers of key cities into single nodes, following administrative boundaries. Then, for each 24 h period, a one was added to a given edge *i*,*j* if a phone moved from node *i* to node *j*. To further filter out intra-city mobility, repeated trips between the same towers for the same user within a 24-period were not counted. This effectively removes “double” trips, and leaves the one-way trips, for example home-work commutes. After all this, mobility was defined and quantified as an asymmetric affinity matrix *A* varying through time, where *A*_*ij*_(*t*) is the total number of unique trips made by users between towers *i* and *j* on day *t* of 2013 (see Fig. 1A for a geographic representation for one of these daily affinity matrices). In order to normalize the data so that the signal from high volume mobility in areas such as Dakar, the capital of Senegal, did not wash out all other signals, we calculated relative mobility by dividing the entries of *A*(*t*), by their respective row sums. In this way the number of trips on a given day from a given node are now weighted by the amount of people moving from that node. In order to perform the SGW analysis, we then converted this asymmetric affinity matrix to a symmetric one by taking the average of two-way mobility patterns between pairs of cell-towers. Last, so as to not include intra-city traffic, we set the diagonal of *A* to zero. The result was a symmetric affinity matrix/network with zeros on the diagonal for every day of 2013.

### Multiscale analysis using spectral graph wavelets

Networks of human mobility and communication are naturally modeled by graphs, sets of nodes connected by edges, each with an associated weight representing the strength of the connection. Here, higher edge weights indicate a greater flow of human migration between the edge’s two endpoints. Graphs can be viewed as discrete analogs of smooth objects such as geometric manifolds and surfaces, and the geometric content of these graphs is often analyzed using an associated Laplacian operator (Chung, 1997; Coifman & Lafon, 2006), as it encodes the structure of the graph in a natural way. In particular, its eigenfunctions can be used to construct a family of wavelets, which allows one to decompose functions in the same spirit as the indispensable Fourier analytic approach to time series analysis. This is the starting point of our study.

In Fourier analysis, one uses eigenfunctions {ϕ_*k*_} of the Laplacian as basis vectors and their associated eigenvalues {λ_*k*_} inform us of the functions’ features: larger eigenvalues are associated to higher frequency or, equivalently, smaller scale variations, and lower eigenvalues correspond to the large-scale features. These functions ϕ_*k*_ are, however, supported across the entire domain in question, and so a function’s Fourier coefficients $c_k = \langle {\rm{\phi}}_k, f \rangle$, integrate features from disparate regions in the domain. Wavelet families, on the other hand, allow one to perform a similar analysis while restricting attention to a local region defined by a central point and a scale, or radius.

Given that human mobility networks are inherently multiscale as well as highly heterogeneous, a method is required that is both multiscale and localized. In the classical settings of time series analysis and image analysis, wavelets were developed to exhibit precisely these two traits. Here we employed a generalization of wavelets to graphs, called spectral graph wavelets. Spectral graph wavelets have been previously used to study human mobility, for example automobile traffic (Mohan et al., 2014), in analyzing human mobility from photo activity via Flickr (Dong et al., 2013), and in general clustering and community detection (Tremblay & Borgnat, 2013).

An important first choice to make when employing spectral graph wavelets is the shape of the wavelet to be used, or in other words the “wavelet kernel". This can have a profound effect on the analysis. In particular, we employed a wavelet kernel based specifically on the heat kernel yielding what we call Hermitian Graph Wavelets (for details of the mathematical analysis see: Gelbaum, Titus & Watson, 2019), which allows us to associate explicit radii to the wavelets rather than a scale parameter that is independent of any metric on the graph. By using these wavelets, we were able to produce a data analysis method that efficiently extracted key geometric information from the time-varying human mobility networks *A*(*t*).

This approach provides a decomposition whose components may be ordered by importance. Whereas in a Fourier decomposition the largest coefficients *c*_*k*_ indicate the eigenfunctions which contain most of the original function’s information, in the present study the norm of the wavelet gives a measure of the gross data encoded by it. The outputs of this analysis are a set of wavelet functions associated to each vertex (i.e. for every cell tower in Senegal), as well as a single *dominant scale* for each vertex representing the scale containing the most information for that vertex. By fixing a choice of vertex and observing how the wavelet at the dominant scale evolves over time we have a vastly simplified geometric summary of how the graph structure is changing near the focal vertex. As a result, these wavelet functions are rich in multiscale geometric content, and we used them to identify and characterize different forms or modes of human mobility that occur throughout Senegal over the year 2013.

### Applying spectral graph wavelets to mobility networks

Given the set of daily affinity matrices, *A*(*t*), the application of Spectral Graph Wavelets is as follows. First, for each of the daily affinity matrices, we constructed their associated Laplacian matrices:

$$\Delta(t) = D(t)-A(t)$$

where $D{(t)_{ii}} = \sum\nolimits_j {(A(t))_{i,j}}$ and *D*(*t*)_*ij*_ = 0 for *i* ≠ *j*. Once done, the eigenvectors and eigenvalues of δ(*t*), {ϕ_*k*,*t*_} and {λ_*k*,*t*_} respectively, were then computed. Then, Hermitian graph wavelets were formed as:

(1) $${\rm{\psi}}_{s,x}(y)=\sum_k s{\rm{\lambda}}_k(t)e^{-s{\rm{\lambda}}_k(t)}{\rm{\phi}}_{k,t}(x){\rm{\phi}}_{k,t}(y)$$

for a chosen set of scales *s*∈{*s*_*n*_} (note that ψ_*s*,*x*_(*y*) = ψ_*s*,*y*_(*x*)). These functions exhibit several properties that make them ideal for decomposing signals measured on large and complex networks: they are localized, in that they provide information for every node, and the power of each wavelet (i.e., its norm, as a vector) gives us a measure of its importance with respect to the global network structure (Gelbaum, Titus & Watson, 2019). This is analogous to classical Fourier analysis where large Fourier coefficients in the decomposition of a function indicate the major modes comprising the function. Rather than forming an orthonormal set, the wavelet functions {ψ_*sn*,*x*_} form a *frame* and rather than the classical Parseval equality, an approximate Parseval equality holds: for a function *f* on the network there are constants 0 < *B* < *C* < *∞* with

$$B\|f\|^2 \leq \sum_{s_n,x}|\langle f,{\rm{\psi}}_{s_n,x}\rangle |^2 \leq C\|f\|^2$$

where

$$\|f\|^2=\sum_x|f(x)|^2$$

with the sum taken over all nodes *x* in the network. The values of *B* and *C* depend both on choice of wavelet kernel and the scales chosen. It is always possible to choose scales and (by renormalizing if necessary) achieve a frame with the values of *B* and *C* as close to 1 as desired (Hammond, Vandergheynst & Gribonval, 2011). The determination of the scales {*s*_*n*_} input into the algorithm is largely ad hoc and some trial and error is required. While it is up to the investigator to make this choice, some general points can be made: the chosen scales should always be positive and the largest should not be too much bigger than the largest eigenvalue. The goal is to get a good partitioning of the interval [0, max {*λ*(*t*) }] relative to the spacing of the eigenvalues, but as the eigenvalues are in general not uniformly spaced some experimentation may be required to find the resolution that is most informative. Having a well distributed set of scales will also ensure that the calculation of wavelet power is not sensitive to small changes in the network’s structure or the specific scales chosen. In other words, calculations will be stable.

With an appropriately chosen set of scales, if we let *f* be a delta function at node *z*, *f* = δ_*z*_(meaning *f*(*z*) = 1 and *f*(*x*) = 0 for all other nodes *x* ≠ *z*), the above Parseval bounds and ψ_*s*,*x*_(*y*) = ψ_*s*,*y*_(*x*) symmetry imply that

$$1=\|f\|^2\approx\sum_{s_n,x}|\langle f,{\rm{\psi}}_{s_n,x}\rangle |^2=\sum_{s_n}\sum_{x}|{\rm{\psi}}_{s_n,z}(x)|^2=\sum_{s_n}\|{\rm{\psi}}_{s_n,z}\|^2$$

Thus the values of ||ψ_*sn*,*z*_||^2^ serve to indicate the relative importance of each scale with respect to the vertex *z*. In this way, large values of ||ψ_*sn*,*z*_||^2^ correspond to the major scales of importance at the node *z*. We utilize this intuition and define the dominant scale at each vertex to be

(2) $$S(z,t) = {\rm arg\; max}_{s_n} \| {\rm{\psi}}_{s_n, z} \|^2$$

This measures the scale at which a given vertex is most well-connected to the rest of the network.

The above Hermitian graph wavelet functions yield a multiscale analysis at each vertex in the graph (i.e., for every cell tower in Senegal). To highlight the ability of spectral graph wavelets to identify multiscale patterns, we chose to build our wavelets with a fixed base point at the vertex corresponding to the city of Touba (denoted *x*_*T*_), and track the scale of the dominant wavelet function *S*(*x*_*T*_,*t*) as above, through time. We abbreviate this as *S*(*t*). We thus obtain a sequence of dominant wavelet functions on the network ψ_*S*(*t*),*xT*_(*t*,*y*), where the first argument indicates the dependance on the changing network structure encoded in *A*(*t*) and the second argument *y* corresponds to vertices of the graph on which the function takes its values.

## Results

**Figure 2:**
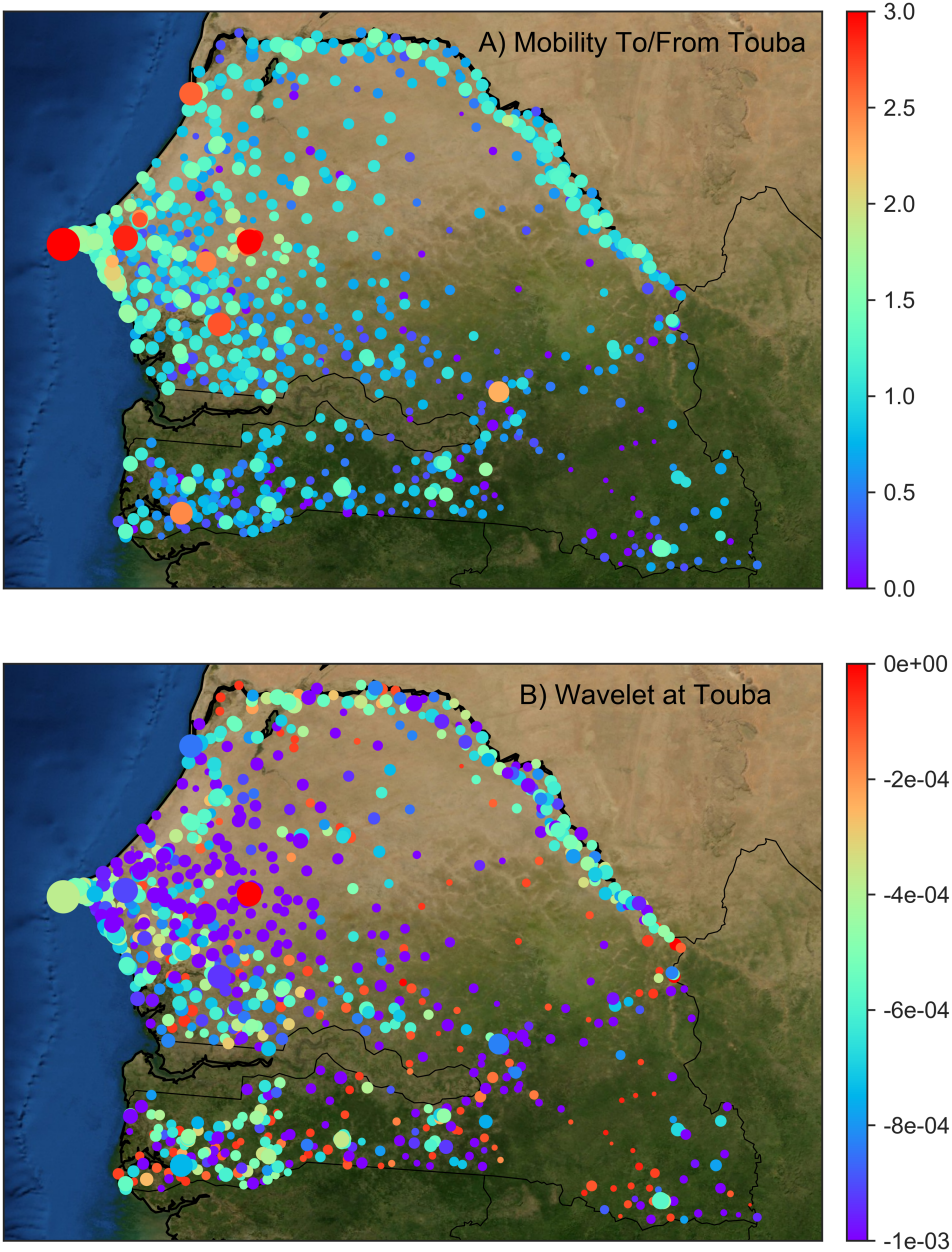
(A) Cell-towers (i.e., human mobility nodes) in Senegal color coded by the log10 number of people moving to/from Touba on a random day in 2013, and sized by local population density. (B) In contrast, colors now denote the dominant wavelet function centered on Touba for the same day. Here, node size remains proportional to local population density. The two maps reveal very different information. In (A) mobility to/from the major urban hubs, such as Dakar the capital, are identified and in (B) the shape of the dominant wavelet function is highlighted: wavelet function values are positive at Touba (the large red node near the center of Senegal) decreasing to large negative values in nearby towns, before becoming less negative at far-off towns).

To demonstrate the utility of spectral graph wavelets for identifying the main spatio-temporal scales and patterns of human mobility, we calculated dominant wavelet functions centered on Touba, the market city in Senegal’s central breadbasket and an important site for religious festivals, and compared results with those produced from a basic analysis of the original population flow data. More specifically, for any given day, human mobility to/from a given location can be extracted from the mobilitymatrices *A*(*t*) and visually inspected (Fig. 2A). Doing so for Touba, one finds that large cities such as Dakar on the west coast account for most of the total daily flow in and out of Touba (i.e., compare the location of large red nodes in Fig. 2A).

In contrast, Fig. 2B depicts the dominant wavelet function for the same day as plotted in Fig. 2A. The function ψ_*S*(*t*),*xT*_(*t*,*y*) revealsa distinct spatial pattern, with a large positive value centered on Touba, that rapidly diminishes to negative values in surrounding nodes, before approaching zero at geographically remote nodes. We note that despite the fact that the wavelet function was calculated using only the population flow data, and not any explicit spatial distance between location pairs, the dependance of human traffic on the distance traveled (i.e., the proximity of Touba to other locations) is apparent in the resulting wavelet function. In other words, we are observing a strong spatial autocorrelation in human mobility levels, which is both intuitive and expected.

**Figure 3:**
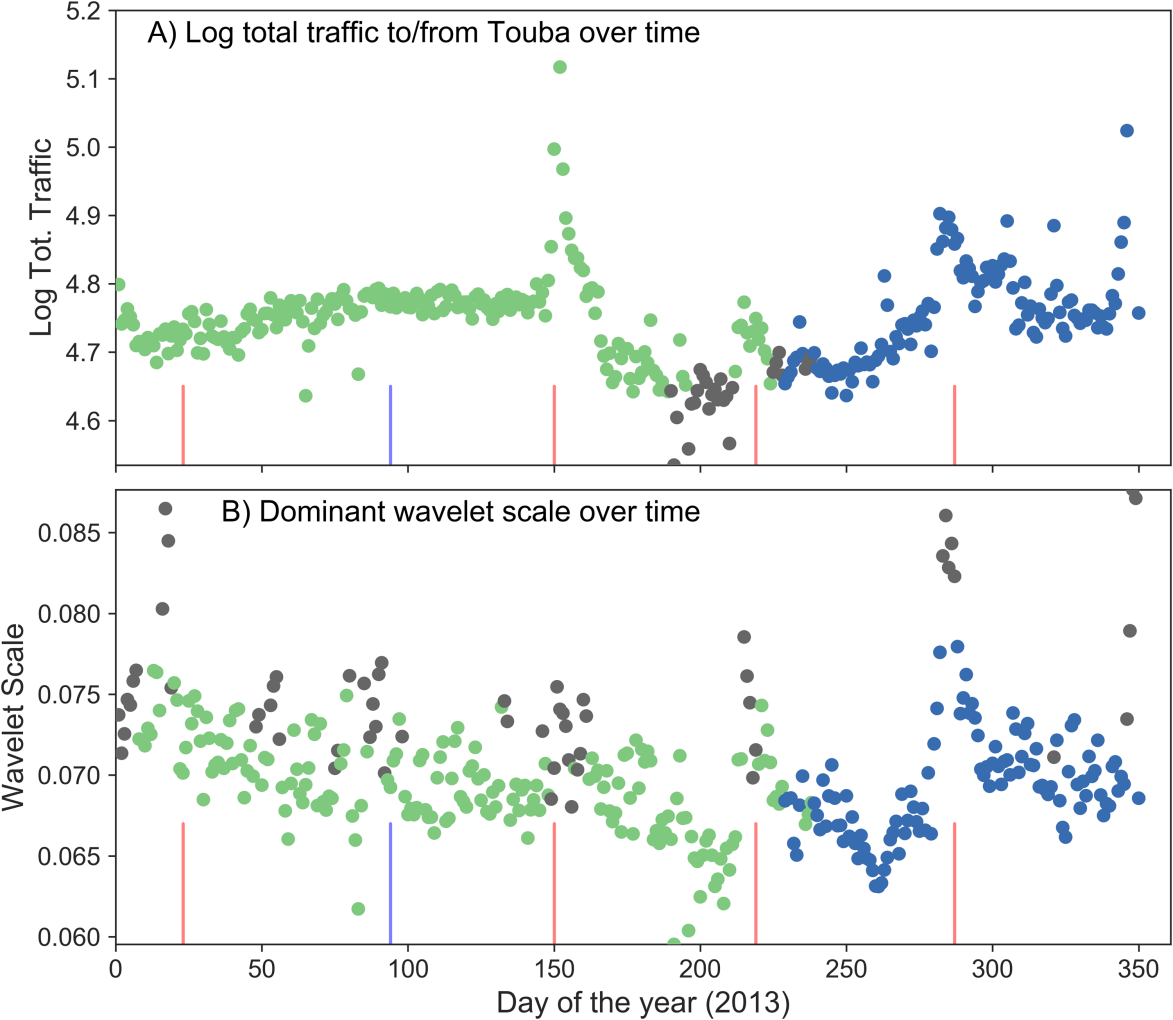
(A) Time-series of the log10 total mobility to/from Touba for 2013, colored by group ID produced from clustering days based on their mobility values. This cluster analysis identifies mobility associated with the dry (green) and wet (blue) seasons (with an transitionary phase in gray), and there are peaks in total traffic to/from Touba that correspond with major religious events at day 150, 219 and 287; however, the cluster analysis does not recognize these events as distinct. (B) In contrast, changes over time in the scale associated with the dominant wavelet function (centered on Touba) better reveals the punctuated and large-scale mobility events related to religious celebrations (vertical red lines) and Senegal’s independence day (blue vertical line). In addition, using these dominant wavelet functions to cluster days extracts meaningful information about seasonal migration (points in green and blue), as well as the short-term/large-spatial scale events (points in gray).

To characterize changes in human mobility through time, focusing on Touba, we first examined changes in the total traffic to/from Touba over time (Fig. 3A); this is calculated by summing the mobility values to/from Touba at all locations for each day (i.e., taking a row-sum of a given affinity matrix *A*(*t*)). *Log*_10_ total mobility to/from Touba over time reveals a variety of qualitative features, most striking is a large peak in traffic corresponding to the commemoration of Touba’s mosque’s 50th anniversary, which occurred May 30th, 2013 (day 150), as well as the end of Ramadan August 7th, 2013 (day 219) and Eid al-Adha on October 15 (day 287).

We then clustered days based upon origin-destination flow values in *A*(*t*). For each day the vector of mobility values to/from Touba were extracted from the mobility matrices *A*(*t*). We clustered days based on these values, using the Louvain method for community detection (Blondel et al., 2008). This involved calculating the Euclidean distance between days based on their mobility values, and running the algorithm numerous times because it is non-deterministic. We chose the Louvain method primarily because it provides an objective determination of the number of clusters. This is in contrast to other common clustering approaches which require the number of clusters to be chosen (e.g., *k*-means). A sensitivity test of the Louvain method, as well as other community detection/clustering approaches is provided in the Supplemental Material.

Clustering these mobility data for Touba identified two main time periods, corresponding to seasonal changes in the Senegalese weather and agriculture, as the rainy season begins around September. There is a third cluster corresponding to a short intermediate period (see Fig. 3A, changes in the marker color: green, gray and blue correspond to the dry, intermediate and wet seasons respectively). Interestingly, the clustering only picked out these long-term changes in mobility, and not the short-punctuated events relating to religious festivals or political events. This is because while these kinds of short-term events are associated with a change in total traffic to/from Touba, any topological changes in the inter-city traffic profile are obscured by the complexity and irregularity of the data.

In contrast to these results created by analyzing the raw mobility values, clustering dominant wavelet functions over time revealed more nuanced information about mobility in Senegal in 2013. Clustering was done using ψ_*S*(*t*),*xT*_(*t*,*y*) in place of population flow values: we calculated the Euclidean distance between daily pairs of Touba-centered wavelet functions, before using the Louvain community detection algorithm to identify clusters. Like the analysis of the relative population flow values described above, this clustering of daily wavelet functions identifies changes in human mobility relating to the dry and wet season. Importantly however, now the presence of relatively short-term and large-scale events were identified throughout the year. These events are marked by a short-term widening of the wavelet function centered on Touba (in network space), and correspond to the three religious migration events listed above. In addition, other events are identified: the Maouloud/Gamou celebration that occurred on January 23, 2013 (day 23) and Independence Day that occurred April 4th, 2013 (day 94). There are two extra events that this clustering approach identified, around day 50 and at the end of the year. The former is an unknown event; the authors do not know of any political, religious, or cultural gathering that occurred near Touba at that time, though we note that the absence of evidence is not evidence of absence. Indeed, the similarity of the network structure at day 50 to that of other verified major migrations is a strong motivator for further investigation. The latter date, at the end of the year, is likely associated with new-years celebrations/holidays. Crucially, the total traffic to/from Touba does not change during many of these events, and they are not identified by clustering the relative population flow data; the scale of the dominant wavelet functions fluctuate due to changes in connectivity within the network and this manages to distinguish between typical traffic patterns and migration events.

**Figure 4:**
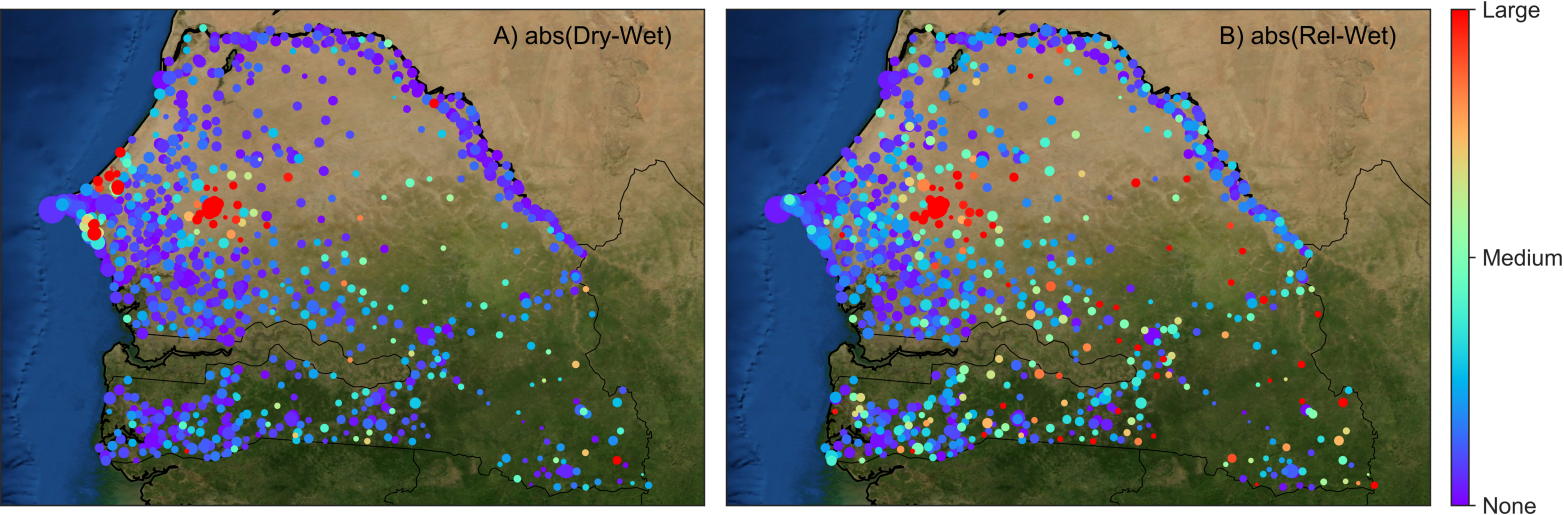
Main spatio-temporal patterns of human mobility can be identified by averaging the dominant wavelet functions associated with the cluster groups shown in Fig. 3B. Then, these modes of human mobility can be compared by calculating their absolute difference and plotting as a map. (A) The absolute difference in the average dominant wavelet function (centered on Touba) associated with the dry and wet seasons highlights changes in mobility to/from the coast and Touba. (B) The absolute difference in the average wavelet function (centered on Touba) associated with religious events and the wet season reveals changes in mobility in far-off towns in the east of Senegal, and Touba.

Averaging the daily wavelet functions associated with each cluster (i.e., producing an average dominant wavelet function centered on Touba for the dry and wet seasons, and for each short-term/large-scale event) reveals stark spatial differences (Fig. 4). For example, the absolute difference between the average wavelet function relating to the dry and wet seasons (Fig. 4A) identifies change in several coastal towns (along with changes in mobility in and around Touba). These changes reflect two processes. First, in Senegal there is a large and mobile agricultural work-force. Seasonally employed farm laborers from the coast use Touba as a stepping stone to rural locations in the interior of the country. Second and also associated with the start of the rainy season are floods, and in 2013 the coast experienced significant flooding. These differences in the average wavelet functions associated with the dry and wet clusters also reflect the impact of the floods on human mobility.

Additional nuances emerge in the punctuated modes of mobility associated with the religious festivals noted earlier. In contrast to the seasonal changes in mobility, the absolute difference between the average wavelet function associated with short-term/large-scale events, in this case Eid al-Adha on October 15 (day 287), and the wet season identifies change in mobility to/from many small rural towns in the far east of Senegal (Fig. 4B). These differences in average dominant wavelet functions clearly identify the long-distance travel that people make as they go to/from Touba for the religious event.

This kind of geographically meaningful information can be used to explore differences in the various modes of human mobility found by clustering daily wavelet functions. In general, this approach more readily reveals heterogenous spatial and network features, relative to results produced from the analysis of the relative population mobility values, which simply described major seasonal changes in flows to/from Touba. Importantly, it bears noting that while we present results from Touba, the spectral graph wavelet analysis also characterizes the main spatio-temporal scales and patterns of human mobility *for every node within the network*. Hence, while we have demonstrated its utility with regards to Touba, there is additional spatially meaningful information that we did not show or analyze.

## Discussion

To improve upon current abilities to characterize changes in human mobility over time we developed and applied a new manifold learning method based on spectral graph wavelets (i.e., Hermitian graph wavelets) to a CDR dataset. These data describe the origin-destination mobility patterns for people living in Senegal, daily for the year 2013. Our approach is non-linear, localized and scale explicit, that is it can be used to identify multiscale patterns of human mobility for each node in a mobility network. This is key to disentangling multiscale patterns with big and rich datasets like those produced by CDRs. Spectral graph wavelets applied to these data allowed us to identify seasonal changes in Senegalese human mobility, as well as punctuated large-scale events relating to religious migration and a national holiday. These short-term but large-spatial-scale events were not identified by a standard approach applied to the original mobility values themselves (i.e., *A*(*t*)), because these data contained too much noise. As a consequence, our spectral graph wavelets approach provides a new method for the multiscale decomposition of human mobility data, and expands the utility of CDR data for anticipating and preparing for changes in human mobility.

Identifying the main spatio-temporal scale and patterns of human mobility, and the spatio-temporal scales at which they occur, is an important part of developing policies for a whole host of operations, ranging from traffic infrastructure to disaster response planning (Jiang, Ferreira & Gonzalez, 2017). These kinds of spatial policies have previously been made using far coarser data, both spatially and temporally (Bell & Ward, 2000). Here, making use of relatively high resolution CDR data, we were able to tease out the spatial signal of punctuated large-scale events, in contrast to a standard approach applied to data on population flow values. The rate at which new data is created—always at higher temporal frequency and greater spatial resolution—is ever growing, and the quantitative tools used to extract useful information from monthly or even annual datasets are rapidly diminishing in utility (Lee & Kang, 2015). For the best use of these new data (i.e., next-gen CDR datasets) new tools are required, and indeed, new tools that can articulate the complex and adaptive nature of human mobility have extreme utility (Scheffer et al., 2018). Like human mobility, other complex adaptive systems are multiscale by nature (Levin, 1998), and in general there is a growing need to extract information about the micro-scale agents that comprise these systems, from which meso- and macro-scale patterns emerge (Folke, 2006). Data-analytic tools designed with the multiscale nature of complex adaptive systems in mind will help policy makers develop plans that explicitly account for the emergence of patterns over a continuum of scales, like in this case the various modes of human mobility in Senegal, and their associated network/geographic scale.

The use of spectral graph wavelets allowed us to essentially transform the origin-destination mobility data (i.e., *A*(*t*)) to a form that better highlights the differences of human mobility patterns. In that spirit, this analysis can be thought of as a process of dimensional reduction or a denoising of the raw data, in a manner that accounts explicitly for network scale. Analyzing the mobility data did not provide the same kinds of scale-dependent information because it is noisy. Admittedly, when analyzing the mobility matrices *A*(*t*) we used a very basic approach to classification. There are indeed many other more sophisticated approaches that we could have been employed, in order to contrast with the results produced from analysis of the dominant wavelets functions. These approaches vary from traditional dimensional reduction techniques that rely on linear correlations, such as Principal Components Analysis (PCA), to machine learning approaches for feature identification (Bi et al., 2003). Indeed, we see that there is a great opportunity for using the wavelet transformed data in combination with machine learning approaches to classification and feature extraction.

The multiscale and localized information that spectral graph wavelets provides can be used in many other ways. Here, we have analyzed human mobility information gained from the CDR data, but the CDR dataset can also be used to construct human communication networks through time. Performing the same analysis on both sets of data would produce concurrent wavelet functions through time. A comparison of changes in the main spatio-temporal scales and models of human communication and mobility might reveal early-warning signals of migration/displacement. Simply put, as people prepare to move they are likely to call their ultimate destination, and this information can help policy makers prepare for changes in population density at specific nodes/places. There is an opportunity to utilize methods from manifold matching (Shen, Vogelstein & Priebe, 2017) to make these comparisons. Manifold matching has been used in image recognition to match photos of the same person, for example. Here, instead of a set of photos from a person’s face, the manifolds that would be compared are those associated with a complex system (the Senegal cellular network) described in two ways (i.e., communications and mobility).

Early detection of large-scale human migration is evident in our analysis. For example in Fig. 3B, the days with large wavelet scale (i.e., the gray dots) often precede the date of the event (i.e., the vertical red lines). This suggest that our method could provide quantitative measures of “anomalous” mobility patterns associated with these events. For example, one could compare a given day’s dominant wavelet function with those from an average wavelet function constructed from the preceding week or month. This is similar to, but contrasts with, what we have done here comparing seasonal patterns of mobility. In doing so, one could compute how anomalous a given day is relative to recent times. This approach to anomaly detection is common, but the use of our HGW method to predict unknown oncoming events from CDR data would be novel.

Additional early-warning signals of mass human-mobility can also be sought from the dynamics inherent to mobility networks alone. These kinds of early-warning signals come from dynamical systems and bifurcation theory (Scheffer et al., 2009) and are measured by changes in the variance and autocorrelation in macroscopic variables (Boettiger, Ross & Hastings, 2013), for example changes in the density of people in a certain neighborhood. For human mobility CDR data, there is an opportunity to advance new early-warning signals of multiscale change using manifold learning. Specifically, spectral graph wavelets is one way to learn changes in the geometry of the manifold on which dynamics occur, but there are others, for example diffusion maps (Coifman & Lafon, 2006) and Laplacian eigenmaps (Belkin & Niyogi, 2003). Systems undergoing a bifurcation driven by some macroscopic variable should *a fortiori* exhibit changes in geometry at small and intermediate scales as well; a multiscale analysis may then allow one to directly address how these kinds of large and abrupt changes in complex systems are related to changes in the behavior of micro-scale agents (i.e. in this case, how individual people move from place to place).

Identifying the main spatio-temporal scales and patterns of human mobility, and potentially early-warning signals of changes between them, is of principal interest of groups tasked with managing human communities (Jiang, Ferreira & Gonzalez, 2017), as they go about their everyday lives as well as respond to infrequent but impactful events like a natural hazard. In Senegal, flooding is a persistent problem and indeed in 2013 the capital Dakar was severely hit. These kinds of events can lead to the permanent displacement of people from their homes, and similarly to identifying the main spatio-temporal scales and patterns of human mobility as done here, there is value to identifying where and when this displacement occurs (Xie et al., 2016). Indeed, displacement is not necessarily instantaneous with regards to the perturbation, but it may take a relatively long time for people to “realize” their displacement (Black et al., 2013). Multiscale methods like spectral graph wavelets applied to CDR data can help distinguish these additional modes of human mobility, and further, methods from manifold matching are likely to be useful too.

In sum, we have made advances to spectral graph wavelets (specifically Hermitian graph wavelets) for analyzing CDR human mobility data. Our approach extracts useful information that is localized and scale-explicit, and we identified seasonal changes in human mobility as well as punctuated large-scale mobility events associated with religious celebrations and a national holiday. Here, we focused our multiscale analysis on one place in Senegal—Touba: a place of religious significance. However, the spectral graph wavelets analysis produces information for all nodes in the network, and there is rich vein of scale-explicit information in the full wavelet transform of the origin-destination mobility data. Last, while the growth in data obtained for complex adaptive systems is daunting (Scheffer et al., 2009), there are opportunities to employ new localized and scale-explicit dimensional reduction techniques, like we have done so here, to greatly improve our ability to characterize and predict multiscale change. This ability is vital if we are to maintain welfare from the complex systems in which we are embedded.

## Supplemental Information

10.7717/peerj-cs.276/supp-1 Supplemental Information 1 Supplementary data and code, including spectral graph wavelet functions.

## Additional Information and Declarations

### Data Availability

The following information was supplied regarding data availability: Data available as a Supplemental File.
