# Supplementary material for: Identifying multiscale spatio-temporal patterns in human mobility using manifold learning
Source: PeerJ Comput Sci. 2020 Jun 15;6:e276. doi: 10.7717/peerj-cs.276 (PMC7924485; doi:10.7717/peerj-cs.276)
Supplement: Supplemental Information 1 [file peerj-cs-06-276-s001.zip › Supp/Supplementary_Infoformation.docx]

SUPPLEMENTARY INFORMATION

**Identifying Multiscale Spatio-Temporal Patterns in**

**Human Mobility using Manifold Learning**

James R. Watson^1^, Zach Gelbaum^1^, Mathew Titus^1,2^, Grant Zoch^1^, and David Wrathall^1^

^1^College of Earth, Ocean and Atmospheric Sciences, Oregon State University

^2^The Prediction Lab LLC, Corvallis, Oregon.

**Supplementary code and data**

The attached python and matlab scripts were used to analyze the raw Call Detail Record data, obtained through the Orange Telecommunications company, specifically their Orange Telecom Data for Development Challenge (D4D). We are not allowed to share these raw data, but more information can be found here:

<https://arxiv.org/abs/1407.4885>

As we are not allowed to share the raw data, we provide aggregated data that were used to make the results/figures presented in the main text. Code used to generate these figures is also provided. We also provide the Matlab code (gwav_*) used to make the spectral graph wavelet calculations. These functions could be applied to other similar data.

In addition to these data and functions, additional methods text is provided below for the sensitivity test performed on the clustering analysis described in the main-text.

**Supplementary Methods – Temporal Clustering Sensitivity Test**

**Introduction**

In order to explore the sensitivity of the temporal clustering conducted using the a) Louvain method (Blondel et al. 2008), which is stochastic, we repeated its application numerous times. We also explored temporal clustering using two other approaches: b) the Newman's spectral community detection algorithm (Newman 2006) applied similarly as the Louvain algorithm and c) using k-means to cluster raw mobility values and dominant wavelet functions alike (this is in contrast to the network community detection methods which were applied to Euclidean distances/similarities computed from the raw mobility values and dominant wavelet functions alike). K-means requires the number of clusters to be chosen. As the Louvain method produced 3 distinct types of days, based on the spatio-temporal patterns of human mobility, we asked k-means to identify 3 clusters too.

**Results**

In the results figures below note that the colors have no meaning other than to distinguish the clusters that the various community detection / clustering algorithms identified.

**
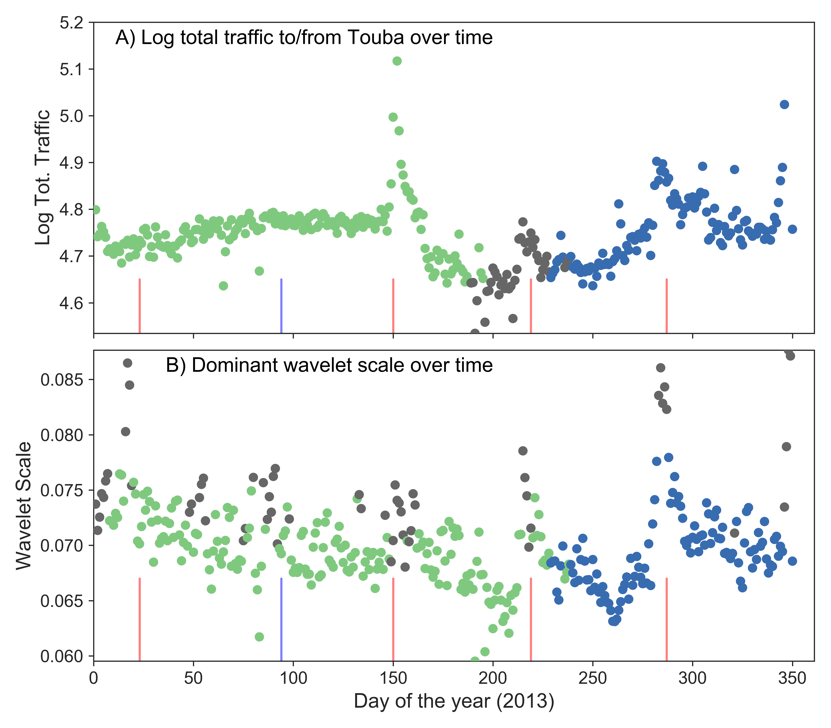
**

**Fig. S1** shows the temporal clustering produced using the Louvain algorithm applied to the Euclidean distances computed between pairs of days based on (A) their raw mobility values and (B) their dominant wavelet functions. This is a second application of the Louvain algorithm in addition to the one whose results are shown in the main test. Even though it is stochastic, these results are identical to those shown in the main text. This indicates that the our overall result -- that grouping dominant wavelet functions reveals various spatio-temporal patterns in human mobility -- is robust.


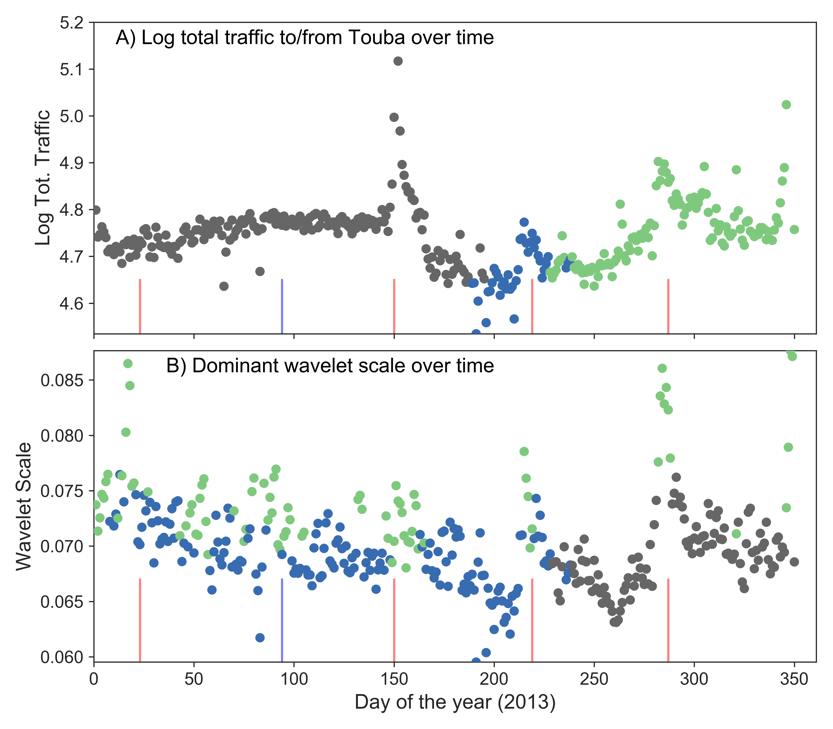


**Fig. S2** shows the temporal clustering produced using the Newman spectral clustering approach, applied to the Euclidean distances/similarities computed between pairs of days based on (A) the raw mobility values and (B) the dominant wavelet functions. The result is very similar to that produced from the Louvain community detection algorithm, albeit with a few slight differences. The main groupings are the same, but a few days are classified differently (for example compare the green cluster of days corresponding to the last red line in Fig. S2 panel B, with its analogue in Fig. S1)

**
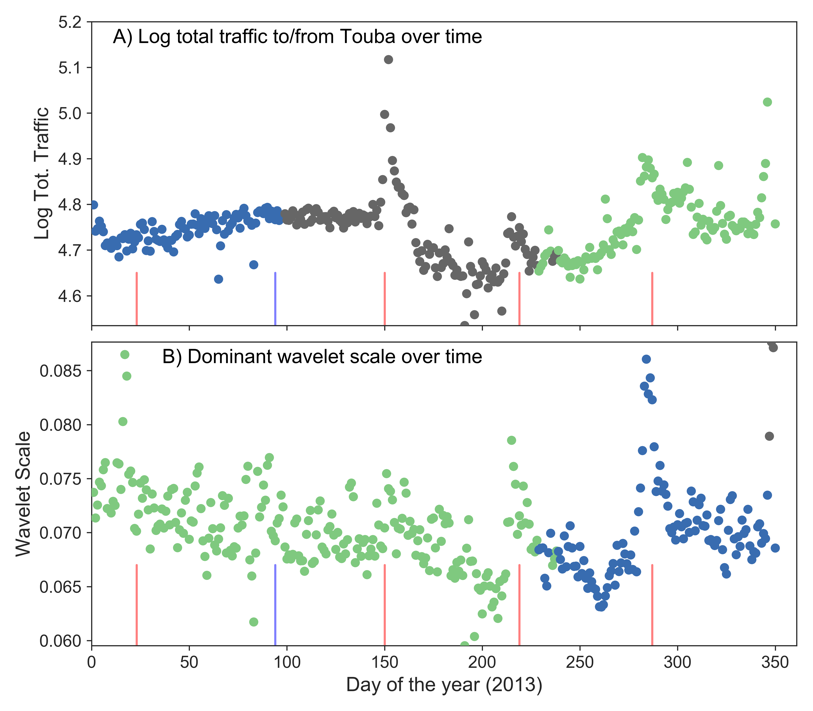
**

**Fig. S3** shows the temporal clustering produced from the application of the k-means algorithm to the raw mobility values (A) and the dominant wavelet functions (B). The k-means algorithm produces very different results to those produced from the Louvain and Newman community detection algorithms. This is a very different approach to grouping days based on the spatio-temporal patterns of human mobility, and it does not capture the changes in the various modes/scales of human mobility as the network community detection approaches. Rather it simply groups days following some rough chronology. This highlights that the type of clustering approach (e.g. modularity optimizing community detection versus k-means) will determine what kinds of information can be gained from the spatio-temporal timeseries analyzed.

**References**

Blondel, V.D., Guillaume, J.L., Lambiotte, R. and Lefebvre, E., 2008. Fast unfolding of communities in large networks. *Journal of statistical mechanics: theory and experiment*, *2008*(10), p.P10008.

Newman, M.E., 2006. Modularity and community structure in networks. *Proceedings of the National Academy of Sciences*, *103*(23), pp.8577-8582.
